# Atorvastatin Can Modulate DNA Damage Repair in Endothelial Cells Exposed to Mitomycin C

**DOI:** 10.3390/ijms24076783

**Published:** 2023-04-05

**Authors:** Maxim Sinitsky, Maxim Asanov, Anna Sinitskaya, Daria Shishkova, Maria Khutornaya, Varvara Minina, Anastasia Ponasenko

**Affiliations:** 1 Laboratory of Genome Medicine, Research Institute for Complex Issues of Cardiovascular Diseases, Kemerovo 650002, Russia; 2 Laboratory for Molecular, Translation and Digital Medicine, Research Institute for Complex Issues of Cardiovascular Diseases, Kemerovo 650002, Russia; 3 Department of Genetic and Fundamental Medicine, Kemerovo State University, Kemerovo 650000, Russia

**Keywords:** genotoxic stress, DNA damage, cytogenetics, micronucleus assay, gene expression, atorvastatin

## Abstract

HMG-CoA reductase inhibitors (statins) are widely used in the therapy of atherosclerosis and have a number of pleiotropic effects, including DNA repair regulation. We studied the cytogenetic damage and the expression of DNA repair genes (*DDB1*, *ERCC4*, and *ERCC5*) in human coronary artery (HCAEC) and internal thoracic artery endothelial cells (HITAEC) in vitro exposed to mitomycin C (MMC) (positive control), MMC and atorvastatin (MMC+Atv), MMC followed by atorvastatin treatment (MMC/Atv) and 0.9% NaCl (negative control). MMC/Atv treated HCAEC were characterized by significantly decreased micronuclei (MN) frequency compared to the MMC+Atv group and increased nucleoplasmic bridges (NPBs) frequency compared to both MMC+Atv treated cells and positive control; *DDB1*, *ERCC4*, and *ERCC5* genes were upregulated in MMC+Atv and MMC/Atv treated HCAEC in comparison with the positive control. MMC+Atv treated HITAEC were characterized by reduced MN frequency compared to positive control and decreased NPBs frequency in comparison with both the positive control and MMC/Atv group. Nuclear buds (NBUDs) frequency was significantly lower in MMC/Atv treated cells than in the positive control. The *DDB1* gene was downregulated in the MMC+Atv group compared to the positive control, and the *ERCC5* gene was upregulated in MMC/Atv group compared to both the positive control and MMC+Atv group. We propose that atorvastatin can modulate the DNA damage repair response in primary human endothelial cells exposed to MMC in a cell line- and incubation scheme-dependent manner that can be extremely important for understanding the fundamental aspects of pleoitropic action of atorvastatin and can also be used to correct the therapy of patients with atherosclerosis characterized by a high genotoxic load.

## 1. Introduction

Mitomycin C (MMC) is a chemotherapy and anti-fibrotic drug used in cancer therapy and various eye surgeries [1,2,3,4,5,6,7,8]. In mammalian cells, MMC undergoes reductive activation to form mitosene [9,10], which reacts via N-alkylation with 7-N-guanine nucleotide residues in the minor groove of DNA followed by DNA crosslinking [11], replication and transcription arrest, and finally, apoptosis [12]. DNA crosslinking can also be triggered by numerous endogenous (byproducts of metabolism) or exogenous (formaldehyde, acetaldehyde, crotonaldehyde, acrolein, pesticides, haloalkanes, alkenes, sulfides, tobacco smoke, exhaust gases, ionizing radiation, etc.) agents [13,14,15,16] that make it possible to use MMC as a model mutagen in genotoxicological studies.

Recently, it was reported that genotoxic stress induced by 6 h of exposure of endothelial cells to 500 ng/mL MMC is associated with proinflammatory activation of endothelium and endothelial dysfunction [17,18,19] underlying atherosclerosis [20,21], a leading cause of cardiovascular morbidity and mortality worldwide [22,23]. HMG-CoA (3-hydroxy-3-methylglutaryl-coenzyme A) reductase inhibitors, also known as statins, are small molecules that are the rate-controlling enzyme of the mevalonate pathway. Statins are widely used in the treatment of atherosclerosis [24] due to their ability to regulate the synthesis of cholesterol and its isoprenoid intermediates, geranylgeranyl pyrophosphate and farnesyl pyrophosphate [25,26]. In addition, statins have a number of cholesterol-independent pleiotropic effects [27,28]. Generally, they can modulate several cellular functions, including DNA damage response, cell homeostasis, proliferation, differentiation, cell survival, and cell death due to the involvement of post-translational modifications of key signaling proteins Ras- and Rho-GTPases [29,30,31,32]. It was shown that statins could trigger apoptosis in tumor cells [33,34], increase their sensitivity to radiotherapy [35,36] and anticancer drugs [34,37,38], and prevent metastatic processes in vivo [39,40]. Statins can also protect various normal cells against cisplatin, doxorubicin, and ionizing radiation-induced damage due to activating JNK/SAPK and NF-kB signaling both in vitro [41,42,43,44,45,46] and in vivo [47,48]. Moreover, statins can promote oxidative DNA damage repair in vascular smooth muscle cells via the stimulation of the NBS pathway [49].

Despite the numerous clinical trials demonstrating the efficacy, effectiveness, and safety of statins, their initiation for the primary prevention of atherosclerotic cardiovascular diseases is debatable due to their relatively uncommon adverse effects (myopathy, new-onset type 2 diabetes, renal and hepatic dysfunction, etc.) [50,51,52]. At the same time, statin therapy is still the first-line treatment for the primary prevention of atherosclerotic cardiovascular diseases in accordance with generally accepted clinical guidelines [53,54].

Given the increasing genotoxic load on the human organism from both environmental and anthropogenic sources, as well as the involvement of genotoxic stress in endothelial dysfunction, the study of the antimutagenic effects of statins is very important for modern biomedicine and vascular biology. The presented research is aimed to study in vitro the modulating effect of atorvastatin against genotoxic stress-induced DNA damage in primary human endothelial cells.

## 2. Results

### 2.1. Results of Cytokinesis-Block Micronucleus Assay

**Figure 1:**
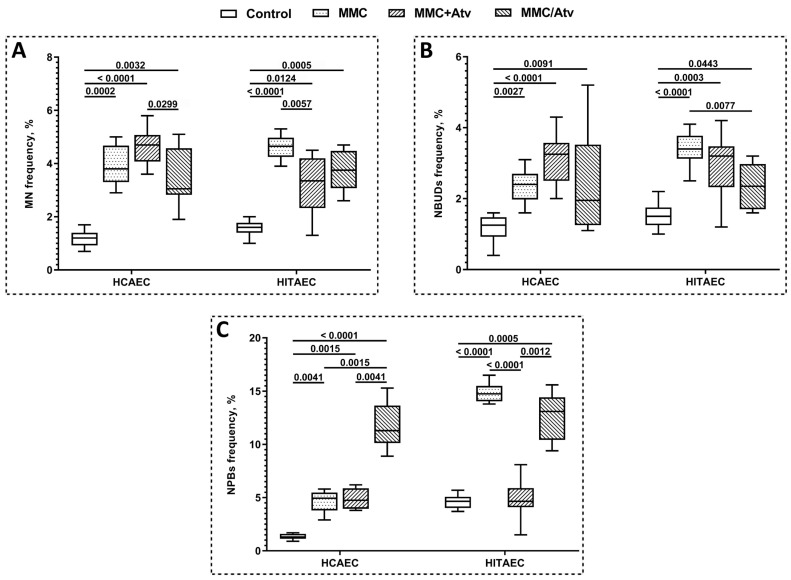
Frequency of MN (**A**), NBUDs (**B**), and NPBs (**C**) in endothelial cells (MMC, mitomycin C; Atv, atorvastatin; HCAEC, human coronary artery endothelial cells; HITAEC; human internal thoracic endothelial cells; MN, micronuclei; NBUDs, nuclear buds; NPBs, nucleoplasmic bridges).

**Figure 2:**
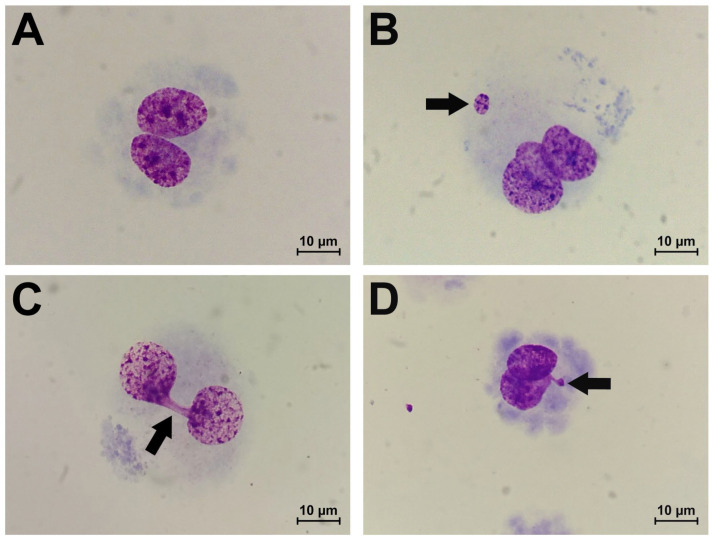
Morphology of undamaged BN endothelial cell (**A**), BN cell with MNi (**B**), BN cell with NPB (**C**), and BN cell with NBUD (**D**) at 1000× magnification (BN, binucleated; MNi, micronuclei; NPB, nucleoplasmic bridge; NBUD, nuclear bud).

The results of the CBMN assay are summarized in Figure 1. The scoring of endothelial cells with MN (Figure 2B) showed that the atorvastatin treatment of HCAEC in both combinations with MMC (MMC+Atv and MMC/Atv) led to no significant modifications (*p* > 0.05) in the MN frequency compared to the MMC-treated cells (positive control).

At the same time, the MMC/Atv treated HCAEC were characterized by a 1.5-fold decreased MN frequency compared to the MMC+Atv group (*p* < 0.05 after FDR correction was applied). In contrast, the MMC+Atv treated HITAEC were characterized by a 1.4-fold reduced MN frequency compared to the positive control (*p* < 0.01) but not to the MMC/Atv group.

We have also shown that NBUDs (Figure 2D) frequency was significantly lower (*p* < 0.01) in the MMC/Atv treated HITAEC (2.4 ± 1.2%) than in the positive control (3.4 ± 0.6%). At the same time, the frequency of this cytogenetic indicator in HCAEC cells from the MMC+Atv, MMC/Atv, and positive control groups did not significantly differ.

The co-incubation of endothelial cells with MMC and atorvastatin led to a 3-fold decrease in NPBs (Figure 2C) frequency in HITAEC (*p* < 0.0001) but not in HCAEC in comparison to the positive control. In the MMC/Atv group, in contrast, the NPBs frequency was significantly (*p* < 0.01) higher compared to both the MMC+Atv group and the positive control in HCAEC and to the MMC+Atv group only in HITAEC.

The frequency of all studied cytogenetic indicators, except the NPBs frequency in the MMC+Atv treated HCAEC, was significantly higher in comparison with the non-exposed control.

### 2.2. Results of Gene Expression Analysis

**Figure 3:**
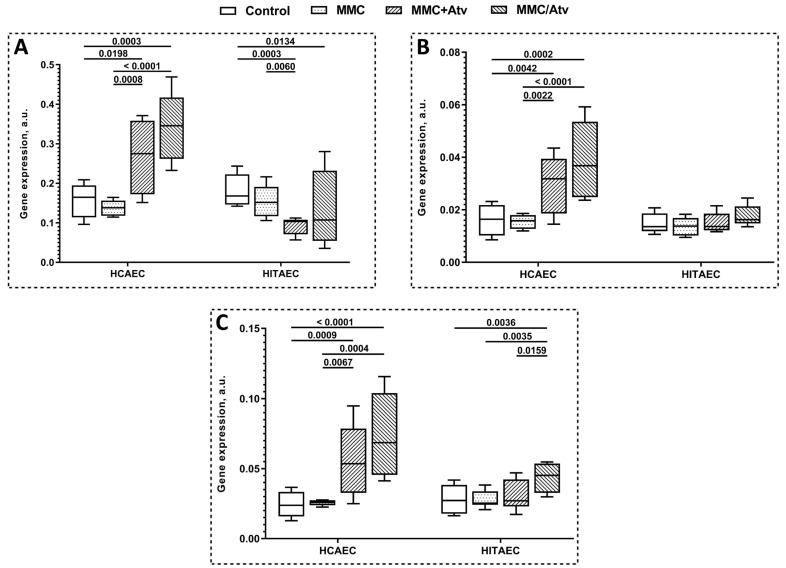
Expression of *DDB1* (**A**), *ERCC4* (**B**), and *ERCC5* (**C**) in endothelial cells (MMC, mitomycin C; Atv, atorvastatin; HCAEC, human coronary artery endothelial cells; HITAEC; human internal thoracic endothelial cells; MN, micronuclei; NBUDs, nuclear buds; NPBs, nucleoplasmic bridges).

The results of DNA repair gene expression profiling are presented in Figure 3. *DDB1*, *ERCC4*, and *ERCC5* genes were upregulated in both MMC+Atv and MMC/Atv treated HCAEC in comparison with the positive control. At the same time, we found no significant differences in the expression of the studied genes between HCAEC treated by MMC and atorvastatin in the different combinations. In HITAEC, the *DDB1* gene was downregulated in the MMC+Atv group compared to the positive control, and the *ERCC5* gene was upregulated in the MMC/Atv treated cells compared both to the positive control and the MMC+Atv group.

## 3. Discussion

Atherosclerosis is a multifactorial inflammatory disease characterized by the accumulation of modified lipids, inflammatory cells, and cell debris in atherosclerotic plaques within the vascular wall [55] and clinically manifested as ischemic heart disease, ischemic stroke, and peripheral arterial disease [23]. According to the various clinical trials, HMG-CoA reductase inhibitors (statins) can effectively prevent the progression of atherosclerosis [56], so they are widely used in clinical practice as the first-line treatment strategy for primary prevention of atherosclerotic cardiovascular diseases [24,53,54]. At the same time, statins have a number of relatively uncommon adverse effects (myopathy, new-onset type 2 diabetes, renal dysfunction, muscle pain and damage, raised blood glucose levels, hepatotoxicity, digestive problems, cognitive effects, and development of rashes and flushing) that make statins therapy debatable, especially in elderly patients [50,51,52]. According to the 2018 ACC/AHA Guideline on the Management of Blood Cholesterol, statins therapy is recommended for secondary prevention of atherosclerosis in older adults (>75 years); however, these recommendations are less direct and rely heavily on patient–physician discussion as well as overall concerns about polypharmacy, frailty, and life-expectancy in case of primary prevention [53,57].

The ability of statins to prevent the progression of atherosclerosis is due to the downregulation of endogenous cholesterol synthesis by the reduction of HMG-CoA to mevalonate [58,59]. Mevalonate is the precursor, not only for cholesterol, but also for a number of nonsteroidal isoprenoid intermediates; therefore, HMG-CoA reductase inhibition results in different pleiotropic effects involved in lipid metabolism and regulation of intracellular signaling pathways [59,60]. It was shown that statins could inhibit the migration and proliferation of vascular smooth muscle cells (VSMCs) and macrophages, downregulate the expression of proinflammatory and proangiogenic cytokines, chemokines, plasminogen activator inhibitor-1, and matrix metalloproteinases in VSMCs and endothelial cells, and improve endothelium-dependent vasomotion and enhance the expression of endothelial nitric oxide synthase (eNOS) [61,62]. Thus, statins can directly affect various cells involved in atherogenesis, including endothelial cells, resulting in preventing endothelial dysfunction—the early event in atherosclerotic lesions [21].

Endothelial dysfunction is triggered by various risk factors, including low or non-laminar shear stress, diabetes mellitus, dyslipidemia, and cigarette smoke [63]. Recently it was reported that DNA damage followed by genotoxic stress could be considered a novel risk factor for endothelial dysfunction [18]. It can be suggested that reducing genotoxic stress in endothelial cells is a promising strategy to prevent endothelial dysfunction and, finally, atherosclerosis.

Atorvastatin is a third-generation synthetic HMG-CoA reductase inhibitor. It was shown that atorvastatin is more efficient in reducing low-density (LDL), very low-density lipoproteins (VLDL), and triglycerides compared to other statins [64,65]. The half-life of atorvastatin is 20 h, which is 5–10 times longer than other HMG-CoA reductase inhibitors [66]. Nowadays, atorvastatin is the most commonly prescribed and widely available in generic formulations of HMG-CoA reductase inhibitors [67]. It was reported that coronary artery disease (CAD) patients are characterized by decreased DNA damage levels measured using the comet assay in peripheral blood lymphocytes after 6 months of atorvastatin therapy [68,69]. Authors have suggested that this effect is associated with the upregulation of high-density lipoprotein-associated antioxidant paraoxonase (PON) after atorvastatin treatment in CAD patients resulting in lipid peroxidation inhibition followed by the reduction of oxidative DNA damage [68]. Moreover, the decreased frequency of BN peripheral blood lymphocytes with MN and NPBs, as well as the number of apoptotic and necrotic cells, was discovered after 3–10 months of atorvastatin therapy in patients with previously untreated dyslipidemia [70]. Thus, the available clinical studies show the ability of atorvastatin to reduce the DNA damage level in patients with CAD and dyslipidemia; however, the mechanisms underlying these effects are still unclear.

Generally, the antimutagenic effects of statins are described for the various cell types (Chinese hamster ovary cells, HeLa cells, primary mouse fibroblast cell line BK4, primary human umbilical vein endothelial cells, human vascular smooth muscle cells) exposed to different triggers of DNA damage, including ultraviolet and ionizing radiation and chemical compounds (cisplatin, doxorubicin, etoposide). It was described that statins involve the inhibition of protein isoprenylation followed by the downregulation of the JNK/SAPK and NF-kB signaling pathways via the inactivation of Ras- and Rho-GTPases [41,42,43]. Statins can also impair DNA strand break formation via the downregulation of the p53 signaling pathway and prevention of checkpoint kinase (Chk-1) activation [44], reduce apoptosis by upregulating thrombomodulin expression and enhancing protein C activation [45], and promote the oxidative DNA damage repair via the stimulation of the NBS pathway [49]. It should be noted that the antimutagenic effects of statins in human arterial endothelial cells have not been studied.

In our experiment, we used the commercially available primary human endothelial cells derived from atherosensitive coronary artery cells (HCAEC) and atheroresistant internal thoracic artery cells (HITAEC) [71,72]. Generally, the atorvastatin treatment of the MMC-treated cells led to the decreasing frequency of MN (acentric chromosome/chromatid fragments or mal-segregated whole chromosomes) and NBUDs (markers of elimination of amplified DNA and DNA–protein repair complexes) [73] in a cell line- and incubation scheme-dependent manner (Table 1). Interestingly, in HCAEC, atorvastatin treatment after MMC elimination (the MMC/Atv group) led to the significantly increased frequency of NPBs formed from dicentric chromosomes as a result of misrepair of DNA breaks or telomere end-to-end fusion [73] compared to the MMC-treated cells, while the simultaneous exposure of cells to MMC and atorvastatin had no effect on the NPBs frequency in comparison to the positive control. Some authors reported that statins in high doses could induce DNA and cell damage. It was shown that atorvastatin at a therapeutic dose of 80 mg/day causes DNA damage in peripheral blood lymphocytes via the generation of reactive oxygen species [74]. Compared to the coronary artery, the internal thoracic artery is characterized by the increasing activity of the endothelial nitric oxide synthase (eNOS) cofactor—tetrahydrobiopterin (BH4). eNOS produces nitric oxide (NO) that has an atheroprotective effect due to the prevention of vascular intima thickening, platelet aggregation, and leukocyte adhesion to the endothelium [75]. Under stress conditions, eNOS undergoes enzymatic uncoupling and becomes an important source of superoxide and peroxynitrite—reactive oxygen (ROS) and nitrogen species (RNS) that can trigger DNA damage. Inadequate availability of BH4 is considered to be an important cause of eNOS uncoupling [76,77]. Therefore, increased BH4 production in the internal thoracic artery leads to reduced ROS and RNS, followed by decreasing oxidative and genotoxic stress in endothelial cells. The reduced level of cytogenetic damage in HITAEC, but not in HCAEC, obtained in our experiment may be associated with the synergistic effect of atorvastatin and the physiologically elevated level of BH4 in endothelial cells derived from the internal thoracic artery.

Several DNA repair systems are involved in the repair of MMC-induced DNA damage: nucleotide excision repair, double-strand break/homologous recombination repair, and the translesion bypass repair pathways [78]. It was reported that *DDB1*, *ERCC4*, and *ERCC5* are the key genes involved in the repair of MMC-induced DNA repair [12]. We propose that in primary human endothelial cells exposed to MMC, atorvastatin can upregulate *DDB1*, *ERCC4*, and *ERCC5* genes that lead to the modulation of DNA damage repair response and decrease genotoxic stress in cell cultures in a cell line- and incubation scheme-dependent manner (Table 1). Interestingly the upregulation of all studied DNA repair genes in HCAEC did not result in a statistically significant reduction in the level of cytogenetic damage; in HITAEC, the downregulation of the DDB1 gene leading to decreased MN and NPBs frequency was discovered, so some mechanisms of post-translation regulation can be supposed. ERCC5 is a protein encoded by the ERCC5 gene involved in homologous recombination repair (HRR) induced by DNA replication stress by recruiting RAD51, BRCA2, and PALB2 to the damaged DNA site. It is known that repair of MMC-induced DNA damage is accompanied by Rad51-recombination complexes that can be detected through the entire nucleus several hours after mutagenic treatment and finally extracted from the nucleus as NBUDs [79]. We can suggest that upregulation of the ERCC5 gene can lead to more effective DNA damage repair in endothelial cells.

It should be noted that molecular mechanisms and pathways underlying the effects of atorvastatin in endothelial cells exposed to alkylating mutagen MMC is unclear and require deciphering. The obtained results can be extremely important for understanding the fundamental aspects of the pleoitropic action of atorvastatin and can also be used to correct the therapy of patients with atherosclerosis characterized by a high genotoxic load. Here, we discovered that atorvastatin, which is widely used in clinical practice, can modulate genotoxic stress in human arterial endothelial cells in an artery-type-dependent manner. This result is important for understanding the fundamental mechanisms of atherogenesis in different arteries. The obtained results also have applied importance in the context of primary prevention of atherosclerotic cardiovascular diseases in regions with a high genotoxic burden on the population (for example, this fact can be considered as an additional justification for prescribing statin therapy to patients).

## 4. Materials and Methods

### 4.1. Cell Cultures and Laboratory Assays

#### 4.1.1. Endothelial Cells Culture

**Figure 4:**
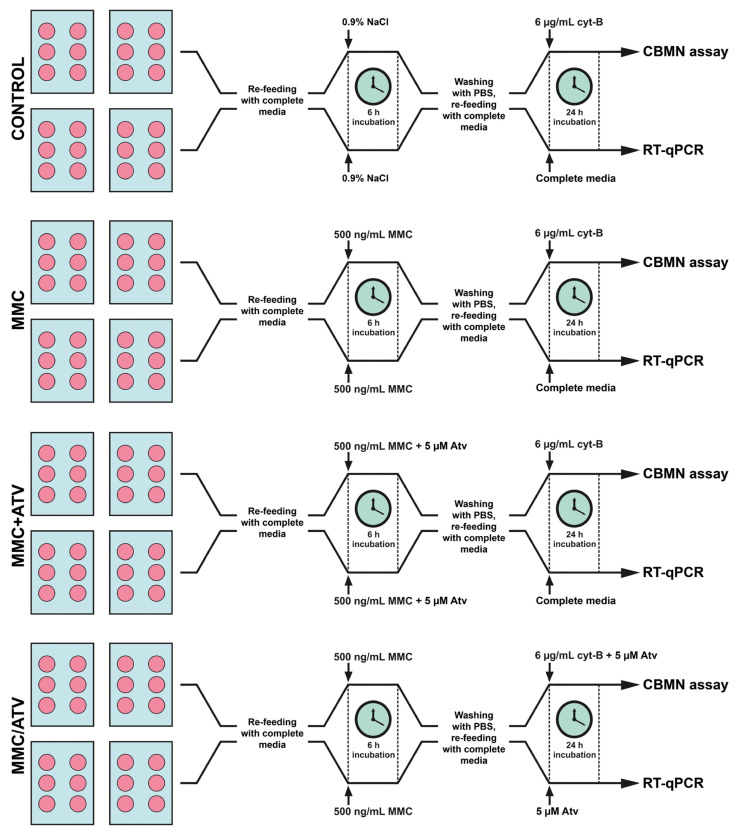
Experimental design of the study (PBS, phosphate buffered saline; cyt-B, cytochalasin B; CBMN assay, cytokinesis-block micronucleus assay; RT-qPCR, quantitative reverse transcription polymerase chain reaction; MMC, mitomycin C; Atv, atorvastatin).

All manipulations with cell cultures were performed in parallel under aseptic conditions. Commercially available primary human endothelial cells derived from atherosensitive coronary artery (HCAEC) and atheroresistant internal thoracic artery (HITAEC) (Cell Applications Inc., San Diego, CA, USA) were plated into fibronectin-coated T-75 culture flasks containing 15 mL MesoEndo Cell Growth Medium (Cell Applications Inc., USA) and cultured at 37 °C, 5% CO_2_, and humidified conditions in an MCO-18AIC CO_2_ Incubator (Sanyo Electric Co. Ltd., Osaka, Japan) until 80% confluency was achieved. Then, cells were trypsinized; 2 × 10^5^ cells were reseeded into 6-well fibronectin-coated plates containing 2 mL of complete media per each well and cultured at 37 °C, 5% CO_2_, and humidified conditions. After 80% confluency was achieved, cells were refed with 2 mL of fresh complete media according to the study design (Figure 4).

In total, two control and two experimental groups for each cell line were formed. HCAEC and HITAEC were exposed to 0.9% NaCl or 500 ng/mL MMC (AppliChem, Spain, CAS no. 50-07-7) for 6 h, followed by 24 h incubation with 6 µg/mL cytochalasin B (AppliChem, Barcelona, Spain, CAS no. 14930-96-2) (downstream cytogenetic analysis) or additive-free complete media (downstream gene expression analysis) were used as the negative or positive control, respectively. In the first experimental group (MMC+Atv), cells co-exposed to 500 ng/mL MMC and 5 μM atorvastatin (Sigma-Aldrich, St. Louis, MI, USA, CAS no. 134523-03-8) for 6 h followed by 24 h incubation with 6 µg/mL cytochalasin B (downstream cytogenetic analysis) or additive-free complete media (downstream gene expression analysis) were included. HCAEC and HITAEC exposed to 500 ng/mL MMC for 6 h followed by the 24 h treatment by 5 μM atorvastatin in complete media (downstream gene expression analysis) or with 6 µg/mL cytochalasin B (downstream cytogenetic analysis) were used as the second experimental group (MMC/Atv). The MMC+Atv group simulated the ability of atorvastatin to prevent new DNA damage, and the MMC/Atv group—the ability of atorvastatin to activate the DNA damage repair.

#### 4.1.2. Cytogenetic Analysis

The level of DNA damage in endothelial cells was assessed using the cytokinesis-block micronucleus assay (CBMN assay) according to the standard protocol [80] with modifications described previously [17]. Cell growth media was removed from each well of 6-well plates; cells were washed with ice-cold PBS, trypsinized by trypsin/EDTA solution, and fixed in 1 mL of methanol/acetic acid (3:1) at −20 °C for 30 min. Then, the cell suspension was centrifuged for 10 min at 1000 rpm, 500–700 µL of the supernatant was aspirated, and the pellet was resuspended and pipetted onto air-dried ice-cold microscope slides. The slides were stained with 5% Giemsa solution for 12 min at room temperature and analyzed using a Zeiss Axiostar Plus Microscope (Carl Zeiss MicroImaging GmbH., Jena, Germany) at 1000× magnification with transmitted light. On each slide, 1000 binucleated (BN) endothelial cells were analyzed; micronuclei (MN), nucleoplasmic bridges (NPBs), and nuclear buds (NBUDs) were scored in these cells according to the generally accepted criteria [80,81].

#### 4.1.3. RNA Extraction

All work surfaces and laboratory equipment used for RNA extraction were treated with RNaseZapTM RNase Decontamination Solution (Invitrogen, Waltham, MA, USA). Cell growth media was removed from each well of 6-well plates, and cells were washed with ice-cold PBS and immediately lysed using 1 mL of QIAzol^®^ Lysis Reagent (Qiagen, Hilden, Germany). Total RNA extracting and genome DNA elimination were performed with the RNeasy^®^ Plus Universal Mini Kit (Qiagen, Germany) according to the manufacturer’s protocol. The quantity and quality of the extracted RNA were evaluated using a NanoDropTM 2000 Spectrophotometer (ThermoScientific, Waltham, MA, USA) by measuring the light absorbance at 280 nm, 260 nm, and 230 nm and calculating the 260/280 and 260/230 ratios. RNA integrity was determined by measuring the RNA Integrity and Quality (RIQ) Index using Qubit RNA IQ Assay Kit (Invitrogen, USA) and the Qubit 4 Fluorometer (Invitrogen, USA). The extracted RNA was stored at −80 °C.

#### 4.1.4. Complementary DNA Synthesis

Complementary DNA (cDNA) was reverse transcribed based on 100 ng of extracted RNA using a High-Capacity cDNA Reverse Transcription Kit (Applied Biosystems, Waltham, MA, USA) according to the manufacturer’s protocol. The quality of synthesized cDNA was assessed using a NanoDropTM 2000 Spectrophotometer (ThermoScientific, USA) by measuring the light absorbance at 280 nm, 260 nm, and 230 nm and calculating the 260/280 and 260/230 ratios. The synthesized cDNA was stored at −20 °C.

#### 4.1.5. Gene Expression Analysis

The expression of key genes involved in the repair of MMC-induced DNA damage, *DDB1* (*XPE*), *ERCC4* (*XPF*), and *ERCC5* (*XPG*) [12], was analyzed with quantitative reverse transcription polymerase chain reaction (RT-qPCR) using the ViiA 7 Real-Time PCR System (Applied Biosystems, USA) and TaqManTM Gene Expression Assays (Applied Biosystems, USA) (Table 2) in accordance with the MIQE Guidelines [82]. The RT-qPCR was performed in 96-well plates containing 26 experimental samples, 5 double diluted standards, and NTC (no template control). Each sample, standard, and NTC were assayed in triplicate. For each analyzed sample, 20 µL of reaction mixture containing 10 µL of TaqManTM Gene Expression Master Mix (Applied Biosystems, USA), 1 μL of appropriate TaqManTM Gene Expression Assay (Applied Biosystems, USA), and 9 μL of cDNA template at a final concentration 50 ng/μL were prepared. The amplification was performed as follows: 2 min at 50 °C, 10 min at 95 °C, 15 s at 95 °C, and 60 s at 60° C (40 cycles). RT-qPCR results were normalized using three reference genes, *HPRT1*, *GAPDH*, and *B2M* [83]. The expression of *DDB1*, *ERCC4*, and *ERCC5* genes was calculated with the ΔCq method. The quality of RT-qPCR was evaluated using the analysis of amplification and standard curves in QuantStudioTM Real-Time PCR Software v.1.3 (Applied Biosystems, USA).

### 4.2. Statistical Analysis

Statistical analysis was performed using the GraphPad Prism 8 software package (USA). The D’Agostino–Pearson normality test was used to verify the compliance of the data with the normal distribution. For quantitative variables, the median (m) and interquartile range (IQR) were calculated. Differences between several independent groups were analyzed with the Kruskal–Wallis test. To avoid the effect of multiple comparisons, the original false discovery rate (FDR) method of Benjamini and Hochberg was applied. Differences were considered statistically significant at *p* < 0.05. Data were graphically presented as a Box and Whiskers Plot displaying the median, 25th, and 75th percentiles, lower and upper extremes.

## Figures and Tables

**Table 1:** Modification of the studied cytogenetic and molecular genetic indicators in endothelial cells in response to atorvastatin treatment compared to the positive control (m ± IQR).

Indicator	HCAEC			HITAEC		
	MMC+Atv	MMC/Atv	Positive Control	MMC+Atv	MMC/Atv	Positive Control
MN frequency, %	4.70 ± 0.80	3.05 ± 1.40	3.80 ± 1.35	3.35 ± 1.75	3.75 ± 1.30	4.65 ± 6.50
NBUDs frequency, %	3.25 ± 1.05	1.95 ± 2.05	2.40 ± 1.65	3.20 ± 1.10	2.35 ± 1.15	3.40 ± 0.60
NPBs frequency, %	4.75 ± 1.85	11.3 ± 3.25	4.95 ± 1.65	1.65 ± 1.85	13.1 ± 3.60	14.75 ± 1.20
*DDB1* expression, a.u.	0.275 ± 0.018	0.346 ± 0.012	0.138 ± 0.033	0.103 ± 0.003	0.107 ± 0.013	0.152 ± 0.063
*ERCC4* expression, a.u.	0.032 ± 0.002	0.037 ± 0.003	0.016 ± 0.005	0.014 ± 0.001	0.016 ± 0.001	0.014 ± 0.006
*ERCC5* expression, a.u.	0.053 ± 0.003	0.069 ± 0.006	0.026 ± 0.003	0.027 ± 0.002	0.045 ± 0.002	0.025 ± 0.007

**Note:** Highlighted in red—upregulated indicators, highlighted in blue—downregulated indicators, highlighted in green—unmodified indicators (in comparison with the positive control).

**Table 2:** Characteristic of TaqMan^TM^ Gene Expression Assays (Applied Biosystems, USA).

Gene	Assay ID	Reporter/Quencher	Assay Design	Amplicon Length
*HPRT1*	Hs02800695_m1	FAM/MGB-NFQ	Probe spans exons	82
*GAPDH*	Hs02758991_g1	FAM/MGB-NFQ	Probe spans exons	93
*B2M*	Hs00187842_m1	FAM/MGB-NFQ	Probe spans exons	64
*DDB1*	Hs01096550_m1	FAM/MGB-NFQ	Probe spans exons	75
*ERCC4*	Hs01063530_m1	FAM/MGB-NFQ	Probe spans exons	64
*ERCC5*	Hs01557031_m1	FAM/MGB-NFQ	Probe spans exons	71

## Data Availability

The data presented in this study are available on request from the corresponding author.
